# Effects of Ectomycorrhizal Fungi and Heavy Metals (Pb, Zn, and Cd) on Growth and Mineral Nutrition of *Pinus halepensis* Seedlings in North Africa

**DOI:** 10.3390/microorganisms8122033

**Published:** 2020-12-19

**Authors:** Chadlia Hachani, Mohammed S. Lamhamedi, Claudio Cameselle, Susana Gouveia, Abdenbi Zine El Abidine, Damase P. Khasa, Zoubeir Béjaoui

**Affiliations:** 1 Faculty of Sciences of Bizerte, University of Carthage, Jarzouna 7021, Tunisia and Laboratory of Forest Ecology (LR11INRGREF03), National Institute of Research in Rural Engineering, Water and Forests (INRGREF), University of Carthage, Hédi Elkarray Street, Elmenzah IV, BP 10, Ariana 2080, Tunisia; chadliahachanii@gmail.com; 2 Center for Forest Studies, Faculty of Forestry, Geography and Geomatics, Abitibi Price Building, Laval University, Quebec, QC G1V 0A6, Canada; mohammed-sghir.lamhamedi.1@ulaval.ca; 3 BiotecnIA, Department of Chemical Engineering, University of Vigo, Rua Maxwell s/n, Building Fundicion, 36310 Vigo, Spain; claudio@uvigo.es (C.C.); gouveia@uvigo.es (S.G.); 4 National Forest School of Engineers, B.P. 5 1 1, Tabriquet, Salé 11015, Morocco; zineenfi@gmail.com; 5 Centre for Forest Research and Institute for Systems and Integrative Biology, Université Laval, 1030 Avenue de la Médecine, Québec, QC G1V0A6, Canada; Damase.Khasa@ibis.ulaval.ca

**Keywords:** ectomycorrhizae, *Pinus halepensis*, heavy metals, plant growth, mineral nutrition, mining, soil remediation

## Abstract

The pollution of soils by heavy metals resulting from mining activities is one of the major environmental problems in North Africa. Mycorrhizoremediation using mycorrhizal fungi and adapted plant species is emerging as one of the most innovative methods to remediate heavy metal pollution. This study aims to assess the growth and the nutritional status of ectomycorrhizal *Pinus halepensis* seedlings subjected to high concentrations of Pb, Zn, and Cd for possible integration in the restoration of heavy metals contaminated sites. Ectomycorrhizal and non-ectomycorrhizal *P. halepensis* seedlings were grown in uncontaminated (control) and contaminated soils for 12 months. Growth, mineral nutrition, and heavy metal content were assessed. Results showed that ectomycorrhizae significantly improved shoot and roots dry masses of *P. halepensis* seedlings, as well as nitrogen shoot content. The absorption of Pb, Zn, and Cd was much higher in the roots than in the shoots, and significantly more pronounced in ectomycorrhizal seedlings—especially for Zn and Cd. The presence of ectomycorrhizae significantly reduced the translocation factor of Zn and Cd and bioaccumulation factor of Pb and Cd, which enhanced the phytostabilizing potential of *P. halepensis* seedlings. These results support the use of ectomycorrhizal *P. halepensis* in the remediation of heavy metal contaminated sites.

## 1. Introduction

Soil pollution is a serious issue, given that it is considered one of the worst environmental problems in North Africa [1,2]. Mining activities are among the major sources of heavy metal contamination in soils [3,4]. Mining generates large quantities of waste, which are disposed of as overburden and tailings near the mine. These wastes are frequently very rich in metallic ores that persist for a very long time [5]. In Southern Mediterranean countries, pollution control plans are very rare and even absent, due to the lack of operational environmental protection laws, and management and pollution control regulations [6]. The Mediterranean climate, which is characterized by hot and dry summers and mild and humid winters, increases the risk of wind transport of contaminated dust and water erosion during infrequent, but generally torrential rain events [6,7]. In North Africa, these mining sites are generally located close to farms that produce foodstuffs for human consumption [6,8,9]. Consequently, both the health of humans and ecosystems are at risk, due to exposure to metal contamination [10,11,12]. Moreover, the spread of contamination can lead to large-scale pollution of agricultural soils, and surface and groundwater sources, thereby leading to potential contamination of the food chain [12,13]. Therefore, the remediation of heavy metal-contaminated sites has become a major concern [14].

Mycorrhizoremediation is one of the most innovative methods that have been recently identified as enhanced forms of phytoremediation [15]. This method relies upon plant–fungal interactions to improve the tolerance and growth of plants in contaminated soils. For several decades, researchers have shown that the use of ectomycorrhizal fungi is a suitable tool for the restoration of mining sites [16,17]. Ectomycorrhizae have been shown to colonize roots under extreme soil conditions [18]. They mitigate metal toxicity in plants by sequestering large quantities of heavy metals [19,20,21]. The fungi can also detoxify metal ions by metal chelation with metallothioneins that can trap contaminants within the Golgi apparatus of their cells [22,23,24]. Furthermore, fungi are able to conjugate heavy metals in various organic molecules, such as glutathione or organic acids [24]. These mechanisms reduce the bioavailability of heavy metals in the soil, and therefore, reducing their uptake by plants, resulting in better plant growth. In addition, ectomycorrhizal fungi are best suited to exploiting metalliferous soils, which are very poor in essential nutrients. In fact, they can improve water-plant relationships and plant nutrition through the extension of their extramatrical phase or extraradical mycelium, together with the massive development of their root lengths and ramifications, which radiate through the soil and facilitate nutrient and water uptake [25,26,27,28,29]. This improved nutrition leads to better health and growth of ectomycorrhizal trees [30].

To facilitate the development of this mycophytoremediation technology, Otero-Blanca et al. [15] recommended the use of free-living fungi. Thus, the use of mycorrhizal seedlings with locally adapted fungi and tree species would appear crucial to improving the survival and growth of seedlings under different stressful site conditions. Different *Pinus* species, which have a wide distribution in the Mediterranean basin, have been encountered in mines in Europe [21]. Previous studies have shown the ability of *Pinus* spp. to form mycorrhizae in North Africa [25,31,32,33]. *Pinus halepensis* Mill. or Aleppo pine is a widespread ectomycorrhizal species [34] that covers 3.5 million ha within the Mediterranean basin [35]; it has been frequently used for the restoration of degraded lands in arid and semi-arid areas [36,37]. This is due to its high tolerance to drought [38], and its ability to grow on calcareous soils [39] and on heavy metal-enriched soils and mine tailings [40,41]. *P. halepensis* has shown great potential to phytostabilize heavy metals [41], together with increasing the efficient use of water and nutrients under adverse soil conditions [42]. Yet, excessive concentrations of heavy metals may exhibit toxic effects, while inhibiting physiological processes of seedlings, including photosynthesis, membrane permeability, enzymatic activity, water balance, and nutrient uptake. In turn, this would induce oxidative stress, and cause growth inhibition and even death [43]. With the decrease in rainfall, and the increasing frequency of drought and extreme temperatures in North Africa, due to climate change [44], the successful restoration of mining sites using tree and agroforestry species remains a major challenge for forest managers. Under these stressful site conditions, the rate of survival of tree seedlings remains very low [45,46]. Our previous studies showed that the production of seedlings that had been inoculated with *Rhizopogon* sp. in modern forest nurseries in North Africa had improved their performance under site conditions [33]. To our knowledge, little is known regarding the use of ectomycorrhizal fungi for the restoration of mining sites in North Africa. The use of mycorrhizal tree seedlings within the framework of restoration and rehabilitation programs for mining sites would certainly help to improve their survival and growth.

When land is disturbed by surface mining operations, the site is subjected to remediation, reclamation, restoration, or rehabilitation, terms that are commonly used interchangeably or otherwise vaguely defined [47]. These definitions range from the avoidance of exposure to pollutants (remediation) to the full recovery of the original ecosystem (restoration). The purpose of the present study is to assess the growth and mineral nutrition of ectomycorrhizal and non-ectomycorrhizal *P. halepensis* seedlings that have been subjected to metal stress. This is done with the aim of future integration of ectomycorrhizal *P. halepensis* seedlings into the rehabilitation of mining sites. In our study, we tested the hypothesis that ectomycorrhizal fungi can enhance the potential of *P. halepensis* in overcoming adverse effects on growth that are posed by a metal-contaminated mining site (Pb-Zn-Cd). In addition, the use of ectomycorrhizal fungi can improve growth and mineral nutrition and give *P. halepensis* seedlings increased tolerance to heavy metals. The current work continues our previous studies on mycorrhization and improvement of the morphophysiological quality of tree seedlings that are produced in modern forest nurseries in North Africa, with a view to increase seedling survival and growth in reforestation sites [32,33].

## 2. Materials and Methods

### 2.1. Experimental Design and Growth Conditions

The experiment was conducted at the National Institute of Research in Rural Engineering, Water, and Forests (INRGREF) in Tunis (Tunisia). The testing experiment is a randomized complete block design (RCBD), with four blocks composed of four treatments: NM-NC (non-mycorrhizal seedlings + uncontaminated soil (control soil)), M-NC (mycorrhizal seedlings + uncontaminated soil), NM-C (non-mycorrhizal seedlings + contaminated soil) and M-C (mycorrhizal seedlings + contaminated soil). A single replicate of each treatment was assigned randomly within a block, and separate randomizations were made for each block. In each block, 20 seedlings were used for each of the four treatments (one seedling/pot), for a total of 320 seedlings (4 blocks × 20 seedlings × 4 treatments). Each pot was filled with the same mass (10 kg) of contaminated or control soil (uncontaminated). The pots were watered to 75–85% of field capacity to prevent leaching of mineral elements and heavy metals.

### 2.2. Sampling and Physico-Chemical Analyses of Contaminated and Control Soils

Contaminated soil samples were collected in the surroundings of the abandoned mine site of “Jebel Ressas” in North Tunisia (36°36′21.4″ N, 10°19′04.0″ E). Eighteen points were randomly sampled to characterize the spatial variability of the soil. The soil was collected from a depth of 20 cm after removing the upper layer. It then was dried at room temperature, passed through a 2-mm mesh sieve, and stored in labeled paper boxes in the dark at room temperature. Several soil samples (for control tests) were taken at points that were located at increasing distances from the Jebel Ressas site following a northwest transect. Pb, Zn, and Cd were analyzed in the samples that were collected from different points. The sample with the lowest concentration (almost zero) of heavy metals was used as uncontaminated soil in the control tests. The control soil was collected, transported, dried, and stored under the same conditions as the contaminated soil. For each soil (contaminated and control soils), three composite samples were formed (six samples per composite sample) and were used to determine mineral nutrients and heavy metal concentrations.

Bulk soil pH (pH_water_ and pH_CaCl2_) was determined according to the method of Rayment and Lyons [48]. In addition to pH_water_, which is commonly used in forest nurseries, pH_CaCl2_ was measured to better characterize and approximate real physicochemical variations within the rhizosphere [48]. Electrical conductivity (EC) was measured after mixing soil and deionized water in a ratio of 1:5 (*w/v*). Total nitrogen and carbon were determined following high-temperature combustion in a LECO analyzer, model CN-2000 (St. Joseph, MI, USA). Heavy metal analysis (Pb, Zn, Cd, P, K, Ca, Mg, Fe, Mn, Cu, B, Na) was performed by Inductively Coupled Plasma-Optical Emission Spectroscopy (ICP-OES) using the model Optima 4300 from Perkin-Elmer (Waltham, MA, USA), as described by Cameselle and Gouveia [49]. All analyses were performed in triplicate, and results were reported as the averages of the three replicates.

After 12 months, soils of the ectomycorrhizal and the non-ectomycorrhizal *P. halepensis* seedlings were analyzed for Pb, Zn, and Cd concentrations. Four composite samples were prepared for each treatment; each composite sample of soil consists of five randomly selected seedlings (five seedlings/block/treatment). The soils were dried at 60 °C for 48 h. Metal concentrations were determined by acid digestion following U.S. Environmental Protection Agency (USEPA) Method 3050B [50], as described by Cameselle and Gouveia [49], using 1 g of dry soil and nitric acid, hydrochloric acid, and hydrogen peroxide. The supernatant was filtered, and metal quantification was determined by ICP-OES (Perkin-Elmer Optima 4300) at the analysis center “CACTI”, University of Vigo (Spain). All analyzes were done in triplicate.

### 2.3. Sampling, Dry Mass Measurements and Plant Tissue Analysis of Ectomycorrhizal Pinus halepensis Seedlings

Plant material consisted of 9-month-old *P. halepensis* seedlings that originated from a forest nursery in northwestern Tunisia (36°57′40.3″ N, 8°59′50.0″ E) and which were grown according to the nursery cultural techniques described by Lamhamedi et al. [32,33,51]. Aleppo pine seedlings were divided into two classes according to the superficial colonization of their root plugs by the extramatrical phase (e.g., extraradical mycelium) of the ectomycorrhizal fungus, as described by Lamhamedi et al. [52]. The first class was composed of seedlings with no superficial colonization of their root plugs by the ectomycorrhizal fungus. The second class included seedlings with root plugs that were covered by the extramatrical phase of the ectomycorrhzial fungus to an area more than 50%. Roots of *P. halepensis* seedlings were colonized by *Rhizopogon* sp. as described by Agerer [53] and Lamhamedi et al. [33]. On a microscopic scale, our observations showed that the structures of ectomycorrhizae are characterized by the presence of mantle hyphae and Hartig net hyphae. This ectomycorrhizal fungus is very abundant and naturally colonizes the seedlings growing in forest nurseries close to forest pine stands in Tunisia [32,33,51].

The initial total dry mass (TDM_0_) of the seedlings was determined using 20 seedlings per class of mycorrhization. TDM_0_ was 2.96 ± 1.40 g (mean ± SD (standard deviation)) and 2.50 ± 0.95 g for mycorrhizal and non-mycorrhizal seedlings, respectively. The seedlings showed no symptoms of mineral deficiency. The initial concentrations of mineral nutrients (N, P, K, Ca, Mg, Fe) and heavy metals (Pb, Zn, Cd) were determined in the shoots and the roots of *P. halepensis* seedlings using three composite samples per class of mycorrhization (five seedlings per composite sample). Likewise, the growing substrate or soil of *P. halepensis* seedlings was subjected to mineral nutrient (N, P, K, Ca, Mg, Fe) and heavy metal (Pb, Zn, Cd) analyses, as described by Cameselle and Gouveia [50], using three composite samples per class of mycorrhization. All analyses were done in triplicate at CACTI, University of Vigo (Spain). Initial characterizations of the seedlings and their growing substrate or soil (mycorrhizal and non-mycorrhizal) are presented in Table 2.

After 12 months of growth, *P. halepensis* seedlings were harvested and washed with tap water to remove soil particles. For each treatment and each block, five seedlings were then randomly selected. Shoots and roots were separated and dried at 60 °C for 48 h to determine to shoot dry mass (SDM) and root dry mass (RDM).

Previously dried *P. halepensis* shoots and roots were milled separately with an electric grinder and kept in labeled plastic boxes. Shoots or roots were mixed to form composite samples, where each consisted of five randomly selected seedlings (shoots or roots) per block and per treatment. Mineral analyses for nutrients (P, K, Ca, Mg, and Fe) and heavy metals (Pb, Zn, and Cd) were carried out as described by Cameselle and Gouveia [49]. The plant samples were first wet-ashed by acid digestion according to the U.S. Environmental Protection Agency (USEPA) Method 3050B [50]. Quantification of nutrients and heavy metals was performed by ICP-OES (Perkin-Elmer Optima 4300). Total nitrogen concentrations were determined on a LECO analyzer (CN-2000) at CACTI, University of Vigo (Spain). All analyses were performed in triplicate (three composite samples). For each sample, mineral nutrient and heavy metal composition was expressed as content (concentration × dry mass) per seedling to accurately reflect seedling heavy metal and mineral nutrient uptake and accumulation [54,55]. The effect of heavy metals on *P. halepensis* seedlings was assessed using the bioaccumulation factor (BAF) and translocation factor (TF) [56], which were calculated as follows:

$$BAF\;=\;\frac{concentration\;of\;heavy\;metal\;in\;plant}{concentration\;of\;heavy\;metal\;in\;soil}$$

$$TF\;=\;\frac{concentration\;of\;heavy\;metal\;in\;shoots\;}{concentration\;of\;heavy\;metal\;in\;roots}$$

### 2.4. Statistical Analyses

Data were analyzed with one-way ANOVA using SPSS 22.0 software (IBM, Armonk, NY, USA) after testing the assumptions of homoscedasticity and normality of residuals [57]. The differences among treatment means regarding all measured variables were determined using Tukey’s tests at a 5% significance level. Each value is presented as the mean ± standard deviation (SD).

## 3. Results

### 3.1. Soil Physicochemical Properties

The initial physicochemical analysis of contaminated and control soils revealed significant differences (*p* < 0.05) between the two soils (Table 1). The contaminated soil had a basic pH (range: 7.73 to 8.87), while the pH of the control soil was circumneutral (range 6.42 to 7.59). As expected, the difference between pH_water_ and pH_CaCl2_ was 1.1 units for both soils. Contaminated soil also exhibited higher electrical conductivity (*p* = 0.0001) and carbon concentration (*p* = 0.0001) than control soil.

Heavy metal analyses revealed significantly higher concentrations of Pb (*p* = 0.0001) and Zn (*p* = 0.0001) compared to the control. In addition, Cd concentration was high in contaminated soil, while Cd was below detection limits in the control soil. The mineral element results revealed higher concentrations for P (*p* = 0.0001), K (*p* = 0.002), Ca (*p* = 0.0001), Mg (*p* = 0.0001), Fe (*p* = 0.001), Mn (*p* = 0.0001), Cu (*p* = 0.046), B (*p* = 0.002) and Na (*p* = 0.002) in contaminated soil compared to control soil. A very high Ca concentration was noted in the contaminated soil, reaching 252 mg·g^−1^ and exceeding concentrations of Pb, Zn, and Cd, which are the main contaminants of the soil.

### 3.2. Growth and Seedling Morphology

**Figure 1:**
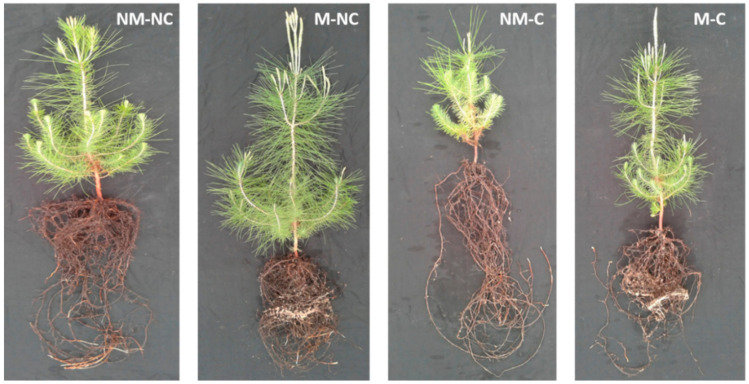
Morphological aspects of ectomycorrhizal (M) and non-ectomycorrhizal (NM) *Pinus halepensis* seedlings after 12 months of growth in contaminated (C) and control soil (NC).

**Figure 2:**
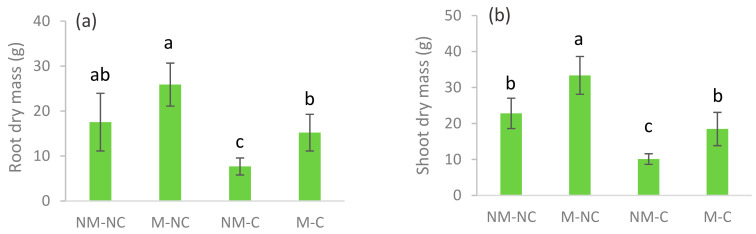
Root dry mass (**a**) and shoot dry mass (**b**) of ectomycorrhizal (M) and non-ectomycorrhizal (NM) *P. halepensis* seedlings after 12 months of exposure to contaminated (C) and control soil (NC). Means (±SD, *n* = 20) with different letters significantly differ from each other based on Tukey’s tests at *p* ≤ 0.05.

The presence of ectomycorrhizae promoted *P.* *halepensis* seedling growth and had positive effects on the performance of seedlings that were grown in contaminated soil (Figure 1). In soil contaminated with heavy metals (Pb, Zn, and Cd), root dry mass (RDM) of *P.* *halepensis* seedlings was significantly reduced (*p* = 0.002) compared to the control (NM-NC). After 12 months, the decrease in RDM of non-mycorrhizal (NM-C) seedlings was 56% compared to the control (NM-NC). In contrast, no significant difference (*p* = 0.792) was found in the RDM of *P.* *halepensis* ectomycorrhizal (M-C) seedlings under contaminated soil compared to the control (Figure 2a). Likewise, shoot dry mass (SDM) was significantly reduced by 55% (*p* = 0.0001) for non-mycorrhizal seedlings in contaminated soil (NM-C) compared to control. Yet, SDM showed a substantial 46% (*p* = 0.0001) increase for mycorrhizal seedlings under control soil (M-NC), while no difference (*p* = 0.698) was reported for M-C compared to the control (Figure 2b).

### 3.3. Mineral Nutrient Contents

Initial characterization of *P. halepensis* seedlings revealed significant variation in mineral element contents between mycorrhizal and non-mycorrhizal seedlings (Table 2). Mycorrhizal seedlings exhibited significantly higher shoot N content (*p* = 0.0001) than non-mycorrhizal seedlings. Moreover, mycorrhizal seedlings had significant lower shoot content for Zn (*p* = 0.0001), Ca (*p* = 0.005), Mg (*p* = 0.004) and Fe (*p* = 0.006). In contrast, higher contents for N (*p* = 0.0001), K (*p* = 0.0001), Ca (*p* = 0.002) and Mg (*p* = 0.004) were recorded in the roots of the mycorrhizal seedlings compared to the non-mycorrhizal ones. Mycorrhizal seedlings had significantly lower root Pb content (*p* = 0.03). The initial growth soil of mycorrhizal seedlings exhibited significantly lower concentrations of Pb (*p* = 0.012), Zn (*p* = 0.005), and Ca (*p* = 0.032) than the growth soil of non-mycorrhizal seedlings. No difference was noted for the other elements (Table 2).

After 12 months, the results of mineral elements in shoots (Table 3) revealed a significant effect (*p* < 0.05) of the toxic metals (Pb, Zn, and Cd). The effect of heavy metal contamination on element content in shoots was more pronounced in NM-C than M-C seedlings. The NM-C treatment resulted in significant depletion of N (*p* = 0.001), P (*p* = 0.042), K (*p* = 0.024), and Fe (*p* = 0.048), while there was no difference between NM-C treatments and control for Ca (*p* = 0.999) and Mg (*p* = 0.628) content. With respect to the control (NM-NC), NM-C showed lower element contents reaching 36%, 56%, 41%, and 28% for N, P, K, and Fe, respectively. In contaminated soil, mycorrhizal seedlings (M-C) exhibited higher contents of N (*p* = 0.0001) and Ca (*p* = 0.012) compared to the control (Table 3). At the end of the experiment, the increase in N and Ca content for M-C seedlings reached respectively 49% and 41% of values for the control seedlings. Further, mycorrhizal seedlings had lower Fe (*p* = 0.045) content in shoots, but there were no differences for P (*p* = 0.694), K (*p* = 0.196), and Mg (*p* = 0.952) between the M-C treatment and the control NM-NC.

Under control conditions, mycorrhizal seedlings M-NC showed an increase in shoot mineral element content for N (*p* = 0.0001), P (*p* = 0.015), K (*p* = 0.006), Ca (*p* = 0.042) and Mg (*p* = 0.045). The mineral element increase in M-NC seedlings, compared to the control, reaching 168% for N, 96% for P, 48% for K, 37% for Ca, and 41% for Mg, after 12 months.

Mineral element content in roots was different from that observed in shoots (Table 3). Prolonged exposure to high concentrations of Pb, Zn, and Cd significantly decreased N (*p* = 0.0001), P (*p* = 0.007), K (*p* = 0.004), Mg (*p* = 0.032) and Fe (*p* = 0.006) content in roots of NM-C seedlings (Table 3). Thus, after 12 months, the decrease in these mineral elements reached 61% for N, 60% for P, 59% for K, 45% for Mg, and 43% for Fe, compared to the control. In contaminated soil, the M-C seedlings showed better performance, with less pronounced reductions of 18% for N (*p* = 0.047) and 36% Fe (*p* = 0.010), compared to the control. Statistical analysis did not reveal differences between M-C and control treatments in terms of P (*p* = 0.504), K (*p* = 0.487) and Mg (*p* = 0.895) root contents. It is important to note that mycorrhizal seedlings (M-C) showed higher concentrations of Ca (*p* = 0.002) than NM-NC treatment.

In control soil, no significant difference was found in roots between mycorrhizal seedlings (M-NC) and non-mycorrhizal seedlings (NM-NC) for K (*p* = 0.462), Ca (*p* = 0.877) and Mg (*p* = 0.840) (Table 3). However, M-NC seedlings had significantly higher contents of N (*p* = 0.001), P (*p* = 0.045) and Fe (*p* = 0.042) in roots than NM-NC seedlings. The increased content of mineral elements in M-NC, with respect to the control, reached 43% for N, 42% for P, and 49% for Fe.

### 3.4. Heavy Metal Content

After 12 months of growth, substantially greater accumulations of Pb, Zn, and Cd had occurred in roots of *P. halepensis* seedlings rather than shoots, especially for M-C and NM-C. Zn was the most important element that accumulated in shoots and roots, followed by Pb and Cd (Zn > Pb > Cd) for all treatments.

**Figure 3:**
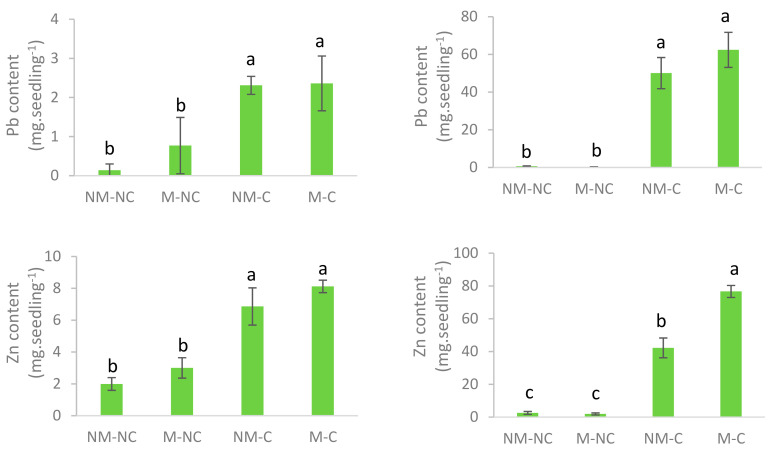

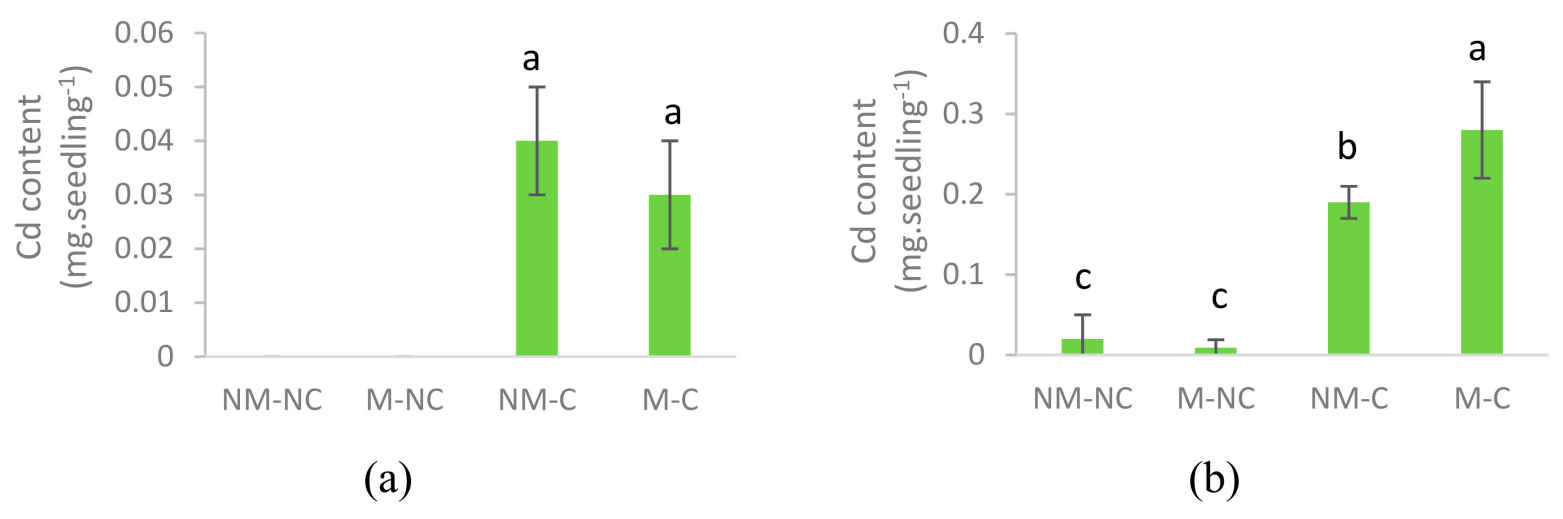
Pb, Zn, and Cd contents in the shoots (**a**) and roots (**b**) of ectomycorrhizal (M) and non-ectomycorrhizal (NM) *P. halepensis* seedlings after 12 months of exposure to contaminated (C) and control soil (NC). Means (±SD, *n* = 3, composite samples), with different letters significantly differ from each other based on Tukey’s tests at *p* ≤ 0.05.

Significant increases in Pb (*p* = 0.004), Zn (*p* = 0.0001), and Cd (*p* ≤ 0.03) contents of the seedling shoots for M-C and NM-C treatments were noted compared to the control. No significant difference was found between NM-C and M-C for shoot Pb (*p* = 0.999), Zn (*p* = 0.226), and Cd (*p* = 0.592) accumulation at the end of the experiment in contaminated soil (Figure 3a). Likewise, Pb, Zn, and Cd root content significantly increased for both M-C and NM-C (*p* ≤ 0.01) compared to the control soil. This effect was more pronounced in the presence of mycorrhizae in the contaminated soil (M-C), but only for Zn and Cd. The increase of Zn root content was 30-fold higher for M-C (*p* = 0.0001) and 17-fold higher for NM-C (*p* = 0.001) relative to the control. Cadmium increased 9.5 times higher (*p* = 0.01) for NM-C and 14 times higher (*p* = 0.0001) for M-C compared to the control. In contaminated soil, mycorrhizal seedlings (M-C) contained higher Zn and Cd than non-mycorrhizal ones (NM-C); these increases were 1.81 times and 1.48 times higher, respectively. No difference was detected (*p* = 0.149) for Pb content between NM-C and M-C (Figure 3b).

Translocation factor (TF) values showed that *P. halepensis* seedlings tend to accumulate Pb, Zn, and Cd in roots rather than in shoots (TF < 1). Mycorrhization reduced Zn and Cd translocation in roots by 31% and 44%, respectively, compared to NM-C. No effect was found for Pb (Table 4). The bioaccumulation factor (BAF) values were lower than 1 for all elements, which indicates their accumulation in the soil rather than in the biomass. The presence of ectomycorrhizae reduced the accumulation of Pb and Cd compared to non-ectomyorrhizal seedlings (NM-C) by 38% and 29%, respectively. No difference was recorded for Zn (Table 4). Soil concentrations of Pb, Zn, and Cd did not vary significantly between NM-C and M-C after 12 months of experimentation (Table 4).

## 4. Discussion

Mine soils are typically extremely stressful and restrictive substrates for plant growth [14]. Initial characterization of contaminated soil revealed high concentrations of heavy metals, high levels of dissolved salts, and a neutral pH (Table 1). These heavy metals have caused a greater decrease in the growth of *P. halepensis* seedlings compared to the control. Nevertheless, this effect was less pronounced in the presence of ectomycorrhizal fungi, which improved the development and growth of the roots and shoots of *P. halepensis* seedlings (Figure 1 and Figure 2). Numerous authors have reported positive effects of mycorrhizal colonization on plant growth and development when they are subjected to mixed metal contamination [23,58,59,60,61]. These studies revealed that mycorrhizal fungi significantly improved the shoot and root growth of inoculated plants. This improvement can be explained by organic acid production [62], greater carbon assimilation [63], and water and mineral supplies [64,65,66,67].

Heavy metal contamination of soil affects plant growth by reducing nutrient availability to plants [68]. At high levels of Pb, Zn, and Cd, our results demonstrated a significant change in mineral nutrient uptake in *P. halepensis* between mycorrhizal and control seedlings (Table 3). Liu et al. [67] showed the effectiveness of mycorrhization on mineral nutrient uptake by plants. These beneficial effects of mycorrhization on plant nutrition vary considerably, according to the mineral element that is involved [69]. Nitrogen in the roots and shoots of mycorrhizal Aleppo pines was significantly higher than that of the non-mycorrhizal pines (Table 3). Liu et al. [67] showed that root colonization increases plant growth and N uptake. Nitrogen and phosphorus concentrations also correlate positively [70]; thus, an increase in N uptake from the soil may result in similar increases in P uptake [71]. Yet, these findings do not entirely agree with our results, where there was no statistically significant difference in shoot and root contents of P compared to controls (Table 3). Similar results were found in *P. halepensis* that were growing on mine tailings in the absence of mycorrhizae; these pines exhibited P-deficiency in the shoots [41]. This response suggests that mycorrhizal presence is crucial to maintaining adequate P acquisition under severe metal contamination of the soil. Plassard and Dell [72] and Plassard et al. [73] found that organic acids released by ectomycorrhizae, such as oxalate, could mediate P acquisition by increasing its availability. Yet, oxalate production is regulated by nitrogen, but this relationship remains poorly understood [72]. In contrast, the high nutrient (N and P) supply level that was provided by the mycorrhizae can improve plant tolerance to oxidative stress [74]. Mycorrhizae also enhance survival and growth by increasing mineral acquisition and plant development [75]. Our findings showed that excess Ca in the shoots of mycorrhizal seedlings (Table 3) decreased Fe and K root uptake at neutral soil pH, as has been described by Lamhamedi et al. [76] under forest nursery conditions. Otherwise, K content in M-C seedlings did not show significant differences, which was further confirmed by the maintenance of K homeostasis. This study underscores the role of mycorrhizal associations in plant K nutrition. At high Pb, Zn, and Cd levels, the ectomycorrhizal symbiosis did not substantially change Mg absorption (Table 3).

All heavy metals in the soil, when at high concentrations, have a strong effect on nutrient absorption [77]. For instance, it has been reported that at high concentrations, Pb binds to ion exchange sites on the cell wall or precipitates in extracellular spaces, thereby blocking the absorption of nutrients [78,79]. Ectomycorrhizal associations with *P. halepensis* significantly affect the adsorption capacity for Pb, Zn, and Cd. Our results showed that the Pb, Zn, and Cd content of roots were much higher than those of shoots, while the roots of M-C seedlings had higher concentrations of Zn and Cd (Figure 3). Similar results revealed that ectomycorrhizae of *Populus × canescens* increased uptake and accumulation of Pb by roots [23]. Moreover, Gu et al. [80] revealed that concentrations of Pb, Zn, Cd, and Cu in roots tended to increase with mycorrhizal colonization. These findings may be explained by a dilution effect, which results from increased plant growth related to a greater capacity to hold nutrients [20,81]. Kong et al. [75] attributed Cd root sequestration to leaf enrichment in N and P. Ectomycorrhizal fungi also seem to trigger metal detoxification in the host plant through the production of organic acids [82]. Metal homeostasis may be maintained by chelation to metallothioneins and glutathione, while cellular compartmentalization [83,84] is promoted by increased expression of metal transporters or by improved management of oxidative stress [85]. Moreover, the mobility of metals in the soil and their availability to plants is closely related to the content and speciation of the element accumulations in plants [86], the organic matter content, and the soil pH. A neutral pH, as noted in the present study, may have further resulted in low metal extractability, while high electrical conductivity could have favored the mobility of heavy metals [41]. Our results showed that mycorrhizae did not have a significant effect on root Pb content (Figure 3). This may relate to the very high concentrations of soil Ca, which saturated ion channels that have a high affinity for calcium, and that are generally taken by Pb for crossing cell membranes [87]. The bioaccumulation and translocation factors were less than 1; the presence of ectomycorrhizal fungi further reduced these values (Table 4), which demonstrates that ectomycorrhizae improved the phytostabilization potential of *P. halepensis*, similar to the findings reported by Gu et al. [80].

## 5. Conclusion and Research Needs

The present work confirmed that the use of mycorrhizae may be a promising approach for increasing growth and mineral nutrient acquisition in *P. halepensis* seedlings. This favors the use of ectomyorrhizal *P. halepensis* seedlings for reforestation programs of heavy metals on contaminated sites in arid and semi-arid zones. With climate change, it would be prudent to acquire a better understanding of the effects of ectomycorhhizal fungi on water relations (water potential, osmotic and turgor potential, modulus of elasticity, etc.) and drought tolerance of *P. halepensis* seedlings in the presence and absence of heavy metals. Nutrient loading using vector analysis would be another avenue to define more precisely the combined effects of heavy metals and ectomycorrhizae on the nutritional status of the seedlings (dilution, sufficiency, deficiency, excess, limiting, non-limiting, antagonistic, and toxic) in response to metallic contamination of soil.

## Figures and Tables

**Table 1:** Physicochemical characteristics of the contaminated and control soils.

	Contaminated Soil	Control Soil
pH_water_	8.87 ± 0.02 a	7.59 ± 0.08 b
pH_CaCl2_	7.73 ± 0.11 a	6.42 ± 0.14 b
EC (µS·cm^−1^)	255.3 ± 3.51 a	152.8 ± 9.25 b
Carbon (mg·g^−1^)	44.300 ± 4.050 a	10.433 ± 1.484 b
Pb (mg·g^−1^)	15.587 ± 0.796 a	0.009 ± 0.0004 b
Zn (mg·g^−1^)	37.766 ± 3.210 a	0.021 ± 0.002 b
Cd (mg·g^−1^)	0.181 ± 0.033	0
N (mg·g^−1^)	<1	<1
P (mg·g^−1^)	0.622 ± 0.042 a	0.084 ± 0.001 b
K (mg·g^−1^)	2.236 ± 0.299 a	0.899 ± 0.073 b
Ca (mg·g^−1^)	252.483 ± 8.765 a	2.076 ± 0.169 b
Mg (mg·g^−1^)	6.034 ± 0.232 a	0.461 ± 0.015 b
Fe (mg·g^−1^)	7.824 ± 0.504 a	4.205 ± 0.559 b
Mn (mg·g^−1^)	0.307 ± 0.004 a	0.070 ± 0.004 b
Cu (mg·g^−1^)	0.036 ± 0.002 a	0.017 ± 0.014 b
B (mg·g^−1^)	0.028 ± 0.002 a	0.016 ± 0.0003 b
Na (mg·g^−1^)	0.694 ± 0.068 a	0.350 ± 0.042 b

Within each row, means (±SD, *n* = 3, composite samples) with different letters significantly differ from each other based on Tukey’s tests at *p* ≤ 0.05.

**Table 2:** Initial characterization of *P. halepensis* seedlings (shoots and roots) and their initial growth soil in the presence and absence of ectomycorrhizal fungi.

	Shoots (mg.Seedling^−1^)		Roots (mg.Seedling^−1^)		Soil (mg.g^−1^)	
Elements	Mycorrhizal	Non-mycorrhizal	Mycorrhizal	Non-mycorrhizal	Mycorrhizal	Non-mycorrhizal
Pb	0.023 ± 0.009 a	0.024 ± 0.002 a	0.007 ± 0.0005 b	0.009 ± 0.0006 a	0.011 ± 0.0008 b	0.016 ± 0.001 a
Zn	0.101 ± 0.0005 b	0.128 ± 0.002 a	0.036 ± 0.001 a	0.034 ± 0.001 a	0.038 ± 0.002 b	0.050 ± 0.002 a
Cd	0	0	0	0	0	0
N	31.641 ± 0.199 a	27.183 ± 0.562 b	6.272 ± 0.148 a	4.725 ± 0.129 b	1.633 ± 0.057 a	1.733 ± 0.057 a
P	4.864 ± 0.097 a	4.675 ± 0.137 a	1.546 ± 0.001 a	1.919 ± 1.061 a	0.265 ± 0.024 a	0.273 ± 0.017 a
K	14.585 ± 0.367 a	13.811 ± 0.459 a	3.238 ± 0.009 a	2.599 ± 0.057 b	1.087 ± 0.066 a	1.167 ± 0.046 a
Ca	12.480 ± 0.132 b	13.644 ± 0.337 a	7.328 ± 0.353 a	5.862 ± 0.116 b	4.700 ± 0.284 b	5.247 ± 0.074 a
Mg	3.848 ± 0.044 b	4.033 ± 0.029 a	1.646 ± 0.045 a	1.469 ± 0.026 b	1.207 ± 0.077 a	1.234 ± 0.106 a
Fe	0.856 ± 0.022 b	1.323 ± 0.081 a	2.496 ± 0.389 a	2.578 ± 0.146 a	8.441 ± 0.676 a	9.319 ± 2.306 a

For each compartment (shoots, roots, and soil), means (±SD, *n* = 3, composite samples) with different letters significantly differ from each other based on Tukey’s tests at *p* ≤ 0.05.

**Table 3:** Mineral nutrient contents (mg.seedling^−1^) in shoots and roots of ectomycorrhizal and non-ectomycorrhizal *P. halepensis* seedlings after 12 months of Pb-Zn-Cd exposure.

		Mineral Nutrient Contents (mg.Seedling^−1^)					
	Treatment	N	P	K	Ca	Mg	Fe
Shoots	NM-NC	178.18 ± 17.87 c	25.37 ± 10.48 b	148.60 ± 26.36 b	187.66 ± 33.11 b	51.18 ± 11.75 b	7.22 ± 0.57 a
	M-NC	477.84 ± 12.39 a	49.69 ± 9.11 a	220.79 ± 16.32 a	258.20 ± 21.59 a	72.43 ± 5.63 a	7.57 ± 1.49 a
	NM-C	114.07 ± 7.65 d	11.20 ± 1.69 c	87.36 ± 3.84 c	185.38 ± 14.58 b	43.21 ± 4.79 b	5.22 ± 0.31 b
	M-C	266.11 ± 5.05 b	32.01 ± 4.39 b	115.44 ± 18.81 b	264.79 ± 15.25 a	54.56 ± 7.75 b	5.47 ± 0.10 b
Roots	NM-NC	143.71 ± 9.69 b	16.20 ± 2.12 b	109.32 ± 13.23 ab	335.64 ± 50.63 b	48.84 ± 9.76 a	50.95 ± 4.48 b
	M-NC	205.14 ± 19.08 a	23.06 ± 4.55 a	128.73 ± 26.61 a	382.70 ± 44.75 b	54.06 ± 9.73 a	75.83 ± 15.49 a
	NM-C	54.78 ± 1.72 d	6.40 ± 0.29 c	45.10 ± 3.73 c	348.78 ± 36.38 b	26.59 ± 2.45 b	29.14 ± 16.42 c
	M-C	117.16 ± 4.36 c	19.24 ± 0.93 ab	90.54 ± 7.74 b	711.18 ± 135.02 a	44.43 ± 6.55 a	32.71 ± 3.24 c

For each compartment (shoots and roots), means (±SD, n = 3, composite samples) with different letters significantly differ from each other based on Tukey’s tests at *p* ≤ 0.05.

**Table 4:** Translocation factor (TF), bioaccumulation factor (BAF), and final concentrations of heavy metals (Pb, Zn, and Cd) in contaminated soil 12 months after commencing the experiment.

	Metallic Element		
	Pb	Zn	Cd
**TF**			
NM-C	0.030 ± 0.006 a	0.124 ± 0.009 a	0.191 ± 0.007 a
M-C	0.031 ± 0.008 a	0.086 ± 0.005 b	0.106 ± 0.004 b
**BAF**			
NM-C	0.161 ± 0.026 a	0.062 ± 0.009 a	0.062 ± 0.009 a
M-C	0.100 ± 0.010 b	0.054 ± 0.002 a	0.044 ± 0.006 b
**Final concentration in soil**			
NM-C (mg.g^−1^)	9.678 ± 0.320 a	27.218 ± 8.839 a	0.116 ± 0.030 a
M-C (mg.g^−1^)	10.128 ± 1.554 a	23.672 ± 4.353 a	0.104 ± 0.007 a

For each metallic element, means (±SD, n = 3) with different letters significantly differ from each other based on Tukey’s tests at *p* ≤ 0.05.
